# Self-harming behaviors among forensic psychiatric patients who committed violent offences: an exploratory study on the role of circumstances during the index offence and victim characteristics

**DOI:** 10.1186/s12888-025-06877-2

**Published:** 2025-04-28

**Authors:** Mark Mohan Kaggwa, Arianna Davids, Heather Moulden, Gary Andrew Chaimowitz, Parwiz Mohibi, Bailea Erb, Andrew Toyin Olagunju

**Affiliations:** 1 Forensic Psychiatry Program, St Joseph’s Healthcare Hamilton, 100 West 5th, Hamilton, ON L89 3K7 Canada; 2 Department of Psychiatry and Behavioral Sciences, McMaster University, St Joseph’s Healthcare Hamilton, 100 West 5th Hamilton, Hamilton, ON L89 3K7 Canada; 3 Mbarara University of Science and Technology, Mbarara, Uganda; 4 Michael G DeGroote School of Medicine, McMaster University, Hamilton, ON Canada; 5 Department of Psychology, University of Waterloo, Waterloo, ON Canada; 6 Department of Psychiatry and Behavioral Sciences, University of Oklahoma College of Medicine, Oklahoma City, OK 73117 USA; 7 Discipline of Psychiatry, The University of Adelaide, Adelaide, SA 5005 Australia

**Keywords:** Forensic, Guilt, Negative emotions, Self-harming behaviors, Shame, Suicidal behaviors, Victims, Violence offences, Weapons

## Abstract

**Background:**

Self-harming behaviors are common among forensic patients with violent index offenses. While various factors, including feelings of shame and guilt, may influence self-harming behaviors, little is known about how the circumstances surrounding the index offense and the victims’ characteristics affect self-harming tendencies among forensic patients. In this study, we examined the association of the circumstances surrounding the index offence and victim characteristics with self-harming behaviors among forensic patients who have committed violent offences.

**Methods:**

The present study consisted of 845 forensic psychiatric patients under the Ontario Review Board who had violent offences (Mean age = 42.13 ± 13.29; 85.68% male) in the reporting year 2014/15. The study examined the association between self-harming incidents with the circumstances during the index offense and victims’ characteristics while controlling for clinical and demographic factors based on multiple hierarchical negative binominal regression.

**Results:**

The prevalence of self-harm was 4.14%, and more than half (61.29%) of the patients with self-harming behaviors had multiple incidents. The total number of self-harming incidences recorded in the reporting year was 113. The results showed that of the overall 24.05% explained by the models, the victim’s characteristics contributed approximately 5% points, and circumstances during the index offence contributed an additional 2% points in explaining self-harming behaviors among forensic psychiatric patients during the reporting year. In the final model, the risk of self-harm increased with having a victim who was a healthcare/support staff or a co-patient/cohabitant.

**Conclusion:**

Self-harm among forensic patients who committed violent offences is associated with various factors, including previous history of self-harm and the victim’s characteristics, especially when the victim was a healthcare/support worker or co-patient. These findings suggest that self-harm might be a maladaptive way of coping with negative emotions, such as feelings of guilt and shame triggered by harming others. Mitigating measures for self-harm among patients with violent offences need to be robust and individualized, taking into consideration vulnerability issues and the best available evidence.

## Introduction

Self-harm is the deliberate infliction of damage to one’s body tissue, regardless of suicidal intent [1, 2]. It is more common among patients involved in the criminal justice system, including those in forensic psychiatric settings, compared to other special and general populations [3–6]. For instance, severe complications of self-harm, especially suicide rates, were 3–8 times higher among the correctional population compared to the general population [7]. The higher rates of self-harm among individuals in the criminal justice system are attributed to a preponderance of individuals with more severe mental health issues in correctional settings, who are often associated with higher levels of self-harming behaviors and suicide [5, 7, 8]. Irrespective of intent, self-harm in forensic psychiatric settings has led to serious injuries, disabilities, and lethal outcomes, partly from using methods that can inflict life-altering injuries [9, 10]. In previous studies, various methods of self-harm, such as hanging, self-poisoning, cutting, choking, jumping, overdosing, and injecting air into their blood, among others, are reported among forensic psychiatric patients [5, 9–12]. Moreover, previous research has underscored a high risk of repeated engagement in self-harm among forensic psychiatric patients, with some patients dying by suicide [5, 6, 9]. Vogel and Verstegen reported that close to half (48.1%) of the patients who engaged in self-harm in the period between 2008 and 2019 had multiple incidents, with a mean of 2.83 (SD = 3.3) and a range of one to nineteen incidents per patient [9].

Various studies among patients in forensic psychiatric settings have suggested multiple factors associated with self-harming behaviors, including sociodemographic factors (such as age, gender, level of education, among others), clinical characteristics (such as younger age at the onset of mental health-related problems, severe psychopathology, higher levels of depression and anxiety, adverse childhood events, emotional abuse, substance use, among others), and forensic related factors (e.g., unfit to stand trial status) among others [6, 9, 12–16]. Many in forensic psychiatry struggle with emotional regulation, leading to violent offenses and self-harm. Emotional dysregulation is strongly linked to these behaviors as a form of coping [17]. Additionally, stress in forensic psychiatric institutions exacerbates self-harm due to perceived restraint, lack of transparency, and feelings of disrespect, while the high-security setting often heightens hopelessness and frustration among patients, an aspect that may lead to self-harming [18].

Notwithstanding, the body of evidence on the risks or factors related to self-harming behaviors among this unique population that keeps evolving and expanding. Of all the risk factors described, a history of self-harming is considered one of the major predictors for an individual to re-engage in a self-harming incident [ [5, 19]. Also, self-harming behavior has a strong but complex link with violent behaviors, including violent behaviors perpetrated by forensic patients [20–26]. The contiguous relationship between violence and self-harm has been explored and described by a unique phenomenon called dual harm [20, 22, 26]. Taken together, there is evidence to suggest that individuals who commit violent offences are at a high risk of engaging in self-harming behaviors and vice versa. With approximately a fifth of forensic patients engaging in dual harm, self-harm becomes a major predictor of the risk of violence to the public [26, 27]. For example, approximately 84% of individuals who engaged in self-harming behaviors had engaged in other types of violence [28]. Therefore, understanding the factors associated with self-harm, including the characteristics of at-risk individuals, other determinants of patients’ psychological response to their offence, and any unique circumstances contributing to repeated incidents of self-harm among the forensic psychiatry population, remains imperative to mitigate risk to others and develop appropriate interventions.

Of note, previous literature has highlighted the potential role of psychological factors in self-harming behaviors in the forensic population [5]. For example, psychological failure to cope with difficult and stressful situations resulting in trouble controlling, expressing, and understanding emotions has been implicated in the risk of self-harming [5, 11, 29]. In addition, exposure to trauma or abuse, either as a victim or a perpetrator, may trigger negative emotions such as guilt and shame, which can motivate self-harm as a maladaptive coping strategy or a form of self-punishment [30, 31]. Notably, the guilt and shame hypothesis suggests that self-harm is a way of regulating these aversive emotional states that arise from perceived or actual wrongdoing [30, 31]. Previous research has shown that forensic patients often experience high levels of shame and guilt after committing an offence, and this can depend on the level of insight, relationship to the victim, or the severity of their index offence [32]. These negative emotions may persist or intensify even after mental health symptoms have improved or as they gain insight into their illness and issues surrounding their forensic status [32].

As shown above, previous research has demonstrated a relationship between negative emotions and self-harm [30]. However, the nuances of this relationship can be further explored. For example, victim characteristics and circumstances surrounding the offence may influence the level of shame and guilt experienced, in turn impacting self-harming behaviors. Research examining the relationship between victim characteristics and circumstances surrounding the offence on self-harming outcomes has not yet been well explored. Hence, this study aims to investigate how the victim’s characteristics (such as age, gender, relationship, and outcome of the victim), as well as the circumstances during the index offence (such as the use of a weapon, intoxication during the index offence, and the number of offences), are associated with repeated engagement in self-harming behaviors among forensic patients. Ultimately, we expect that the findings from this study will contribute to the understanding of the psychological interplays underlying self-harm among forensic patients and inform the development of effective interventions to reduce the risk of self-harm and suicide in this population.

## Methods

### Study population

The present study was completed using data collected on forensic psychiatric patients under the Ontario Review Board (ORB) during the reporting year 2014/15, focusing specifically on those who were involved in violent index offences (i.e., murder - including attempted murder; assault - including assault causing bodily harm and aggravated assault; robbery - including bank, store, and purse snatching; abduction - including attempted abduction; and threatening with a weapon). Forensic psychiatric patients in Canada are a group of individuals with mental illness who are legally found unfit to stand trial (UST) or not criminally responsible for their index offences due to mental illness (NCR) [16, 33–35]. The database was created based on the information captured from reports submitted to the ORB about individual patients managed by Ontario’s 12 forensic psychiatry facilities. In total, 1,233 individuals were captured in the ORB database. However, a total of 845 individuals with at least one violent offence were eligible for the present study.

Previous publications have described the database and provided information about the population [15, 16, 26, 35, 36]. The information in the ORB reports is prepared by healthcare professionals with special training in forensic psychiatry, including forensic psychiatrists, psychologists, social workers, occupational therapists, and nurses. The report provides details about various aspects of an individual within the forensic system during the reporting year, including self-harming behaviors. The reports have similar formats and information; however, the length can significantly vary based on how eventful the year was for individual patients and the need to provide comprehensive descriptions of notable events about the patients during the reporting years. The reports contain full details of patients’ history, with each year’s progress being added annually. The patient’s psychiatrist checks the information in the report and takes full responsibility for the information presented when testifying to the ORB. The hospital administrators and the patient’s psychiatrist signed off on each patient’s report submitted to ORB.

### Data collection process for the information in the database

Highly trained research assistants with extensive knowledge and experience in forensic psychiatry research meticulously extracted data from the ORB hospital reports using a standardized coding form. The training of research assistants was completed by an interdisciplinary team of researchers- experienced psychiatrists, clinical psychologists, and researchers in forensic psychiatry. The coding process was conducted in pairs to ensure accuracy and reliability, with each pair reaching a consensus for every patient through thorough discussions. The principal investigator, GAC, promptly addressed and resolved any ambiguities or unclear aspects, ensuring the highest level of data integrity. The meticulously captured variables were then systematically entered into SPSS for comprehensive data cleaning. Note, for the case of victim characteristics, we captured aspects of the most serious violent offences and prioritized extra-familial relationships over intra-familial relationships when there were more than two victims.

### Variables selected for the current study

To answer the research questions proposed in this study, we included data on the following variables/blocks: (1) Demographic variables (including age and gender); (2) clinical variables (history of self-harming behaviors before the reporting year, primary psychiatric diagnosis, previous hospitalization due to mental illness before their time in the forensic system), and duration since the index offence; (3) victim characteristics (i.e., age, gender, relationship with the patient, the severity of the injury after the index offence); and (4) circumstances surrounding the index offence including the use of a weapon, substance of addiction, and the number of offences.

The primary outcome variable was the number of self-harming incidents per patient during the reporting year. The ORB reports contain information on all self-harming occurrences within the reporting period to ensure the safety of the individuals in the forensic system and inform appropriate resources to manage them. The current manuscript captures the total number of incidents of self-harm as a continuous variable.

### Statistical analysis

The data analysis was performed using STATA version 17.0. The variables included in this study were presented with descriptive statistics, including percentages and frequencies for categorical variables and mean (± standard deviation) or median and interquartile range for continuous variables, depending on their distribution. Due to the nature of the captured data, multiple missing data were present, and the listwise deletion method was applied to address this limitation [37, 38].

Due to the presence of overdispersion of this outcome variable (i.e., the mean being greater than the variance: average = 0.13 and variance = 2.57), the main analysis used a multiple hierarchical negative binomial regression to examine the factors that are independently associated with the number of incidents of self-harming behaviors among forensic psychiatric patients. In the model, the outcome variable was the incidents of self-harming behaviors (numbers/frequencies), and the predictor variables were grouped into four blocks, including (1) patients’ demographic characteristics, (2) clinical characteristics, (3) victim characteristics, and (4) circumstances during the index offence(s). The variables in the blocks are described in the variable Sect. 2.2.

The analysis included four models and involved the addition of one block of predictor variables to the model in hierarchical order based on the proposed hypotheses (further details included below). The χ2 statistic, the *p*-value, and the pseudo-R^2^ assessed the model fit and significance. The effect of each predictor variable on the outcome was measured by the incidence rate ratio (IRR) and the 95% confidence interval (CI), controlling for other variables in the model. All statistics were calculated at a 95% level of confidence and 5% statistical error.

Models 1 and 2 controlled for demographic and clinical characteristics, respectively. Models 3 and 4 added the victims’ characteristics and circumstances during the index offence, respectively. The last two models (using a block of predictor variables that captured information on the victim’s characteristics and circumstances surrounding the index offence) were used to test the data and answer the research question– what is the association of victim characteristics and circumstances during the index offence with self-harm to understand better the statistical contribution of this relationship to the variation in self-harming incidences among forensic patients.

For this analysis, we excluded the victim’s age from the variables considered for the models due to missing values. Time or duration from the index offence until data was captured was considered a clinical characteristic, as it mainly depends on clinical progress and risk management.

## Results

### Characteristics of the patients and victims

Of the 1233 individuals in the ORB database for the reporting year 2014-15, 845 individuals with a violent index offence were included in this study. The patients’ mean age (± standard deviation) was 42.13 ± 13.29 years, and the majority were male (*n* = 724, 85.68%). The most common diagnosis was having a schizophrenia spectrum disorder diagnosis (82.72%), and about 15.28% had a history of self-harm in their lifetime. Regarding the victims of violent offences, the majority were male (47.10%), and adult strangers were most often the target of assaults (28.40%). In total, approximately 46.63% of the patients used weapons during the index offence, and 100 (11.83%) were intoxicated during the period around the index offence. See Table 1 for more details.

**Table 1:** Characteristics of forensic patients whose index offence was violent and their victims

Variable	Additional details on the variables/subgroups	Descriptive statistics *N* = 845 *n* (%)
**Patients’ characteristics**		
Age (years)	Age during the reporting year	Mean 42.13 (Standard Deviation (SD) = 13.29)
Gender (as reported by patient)	Male	724 (85.68)
	Female	121 (14.32)
Previous hospitalization for a psychiatric condition	No	127 (15.03)
	Yes	708 (83.79)
	Unknown	10 (1.18)
Primary psychiatric diagnosis, n (%)	Schizophrenia spectrum disorders	699 (82.72)
	Mood disorders	68 (8.05)
	Others	78 (9.23)
Years since index offence	In years (*n* = 841)	Median = 6 and Interquartile range 3–10
Previous history of engaging in self-harming behaviors	No	693 (82.70)
	Yes	125 (14.92)
	Unknown	20 (2.39)
**Victim characteristics**		
Victim gender, n (%)	Male	398 (47.10)
	Female	367 (43.43)
	Unknown	80 (9.47)
Victims’ age (years)	Age at the time of the offence (*n* = 105)	Median = 36 and Interquartile range = 1–63
Victims’ relationship with patients, n (%)	Stanger	258 (30.53)
	Acquaintance	111 (13.14)
	Friend	16 (1.89)
	First degree relatives (Son/daughter/Sibling)	168 (19.88)
	Lover/partner/spouse	45 (5.33)
	Other/extended family members	24 (2.84)
	Legal authority figure	86 (10.18)
	Healthcare/support staff	68 (8.05)
	Co-habitant/co-patient	23 (2.72)
	Others	10 (1.18)
	Unknow	28 (3.31)
Victim’s injury, severity and outcomes, n (%)	None or minor	525 (62.13)
	Injury requiring hospitalization	190 (22.49)
	Injury resulting in death	85 (10.06)
	Unknown	45 (5.33)
**Circumstances during the index offence**		
Patient was intoxicated during the index offence, n (%)	No	719 (85.09)
	Yes	100 (11.83)
	Unknown	26 (3.08)
Weapon use, n (%)	No	425 (50.30)
	Yes	394 (46.63)
	Unknown	26 (3.08)
Total number of offences		Median = 2, interquartile range 1–11

### Self-harming behaviors

A total of 35 (4.14%) patients had engaged in self-harming behaviors during the reporting year. Four individuals had missing data on the number of incidences during the reporting year. However, among the remaining records, 19 (61.29%) had engaged in multiple incidents of self-harming behaviors, and 12 (38.71%) had only one incident. One patient had an exceptionally high number of incidences in one year (*n* = 44). The average number of incidents among those who had a self-harming incident in the reporting year was 0.13 (SD = 1.60) [variance = 2.57]. Among them, the majority (*n* = 12) had one self-harm behavior incident, followed by 10 patients who had engaged in two self-harming behavior incidents.

### Models for predictors of the number of incidents of self-harm behaviors in patients

Table 2 shows the multiple hierarchical negative binomial regression analysis results with four models to predict the incidence rate ratio (IRR) of self-harm behaviors. Model 1 includes selected demographic characteristics of the patients, which explained approximately 0.2% of the variation in the number of self-harm incidents. In this model, being female was statistically significant, with an incident rate of self-harming 5.02 times higher than that of males. The model’s explanatory power increased substantially by approximately 16% points with the addition of clinical characteristics (model 2). In model (2), the IRR was higher for individuals who had stayed longer in the forensic psychiatric system or criminal justice system, had a history of self-harm before the reporting year, and had other primary psychiatric disorders. Model 3, incorporating victim characteristics, accounted for approximately 22% of the variance in the number of self-harm incidents. This model showed a significant increase in the IRR for the following factors: a history of psychiatric hospitalization and if the victim was a healthcare/support staff or co-patient/cohabitant. However, an increase in the patient’s age in Model 3 was associated with a significant reduction in the IRR. The final model (4) incorporated additional circumstances during the index offence, which enhanced the explanatory power of the included factors for self-harm by approximately 2% points. However, none of the newly added variables related to the circumstances during the index offence(s) exhibited a statistically significant relationship with self-harming incidents. The factors identified as significant in Model 3 retained their statistically significant association with the IRR of self-harming incidents.

From the final model, several factors were associated with an increased risk of having self-harming behaviors, including previous history of self-harming behaviors before the reporting year, having other diagnoses, and if the victim was a healthcare/support staff or a co-patient/cohabitant. However, the risk decreased with an increase in age.

**Table 2:** Factors associated with the number of incidents of self-harming behaviors

Variable		Model 1 (*n* = 841)	Model 2 (*n* = 808)	Model 3 (*n* = 808)	Model 4 (*n* = 693)
		χ^2^ = 6.89	χ^2^ = 63.92	χ^2^ = 68.46	χ^2^ = 74.56
		Pseudo *R*^2^ = 0.0178	Pseudo *R*^2^ = 0.1742	Pseudo *R*^2^ = 0.2197	Pseudo *R*^2^ = 0.2406
		*p*-value = 0.032	*p*-value < 0.001	*p*-value < 0.001	*p*-value < 0.001
		Incidence Rate Ratio [IRR] (95% CI)	IRR (95% CI)	IRR (95% CI)	IRR (95% CI)
Constant		0.17 (0.02–1.40) **	0.11 (0.01–0.79) **	0.09 (0.01–0.81) **	0.09 (0.01–3.45)
Patient demographic characteristics	Age	0.98 (0.94–1.03)	0.94 (0.90–1.00)	0.89 (0.82–0.97) *	0.90 (0.83–0.98) *
	Female gender	5.02 (1.23–20.40) **	0.42 (0.07–2.66)	0.39 (0.05–3.16)	0.42 (0.06–3.08)
Clinical characteristics	Previous hospitalization for a psychiatric condition		0.69 (0.17–2.89)	0.80 (0.14–4.43)	0.64 (0.11–3.81)
	Primary psychiatry diagnosis				
	Mood disorders		0.72 (0.07–6.87)	7.00 e-07 (0 -.)	1.01 e-07 (0 -.)
	Others		27.72 (6.75–113.80) **	10.03 (2.03–49.60)	12.14 (0.11–70.34)
	Previous history of engaging in self-harming behaviors		11.28 (3.65–34.85) **	11.22 (3.00–41.88) **	9.21 (2.33–36.39) *
	Years in criminal justice system since index offence		1.08 (1.01–1.17) *	1.12 (0.99–1.27)	1.12 (0.99–1.26)
Victim characteristics	Victims’ gender female			1.43 (0.37–5.44)	1.23 (0.33–4.53)
	Victim’s relationship				
	Acquaintance			1.27 (0.13–12.33)	0.88 (0.08–9.56)
	Friend			0.96 (0.01–61.67)	0.81 (0.01–86.17)
	First degree relatives (Son/daughter/Sibling)			3.02 (0.50–18.20)	2.73 (042–17.75)
	Lover/partner/spouse			5.26 (0.35–79.23)	3.88 (0.25–61.26)
	Other/extended family members			3.42 (0.16–72.65)	3.43 (0.14–81.30)
	Legal authority figure			2.74 (0.22–32.97)	2.23 (0.17–28.41)
	Healthcare/support staff			35.43 (3.89–322.89) *	32.69 (3.41–313.62) *
	Co-habitant/co-patient			121.13 (6.52–2250.23) *	67.01 (4.04–1114.63) *
	Others			4.99e-07 (0–.)	4.29 e-08 (0 -.)
	Victim injury severity and outcomes				
	Injury requiring hospitalization			2.71 (0.61–11.97)	1.98 (0.38–10.29)
	Injury resulting in death			2.93 (0.35–24.58)	2.32 (0.27–19.53)
Circumstance during the index offence	Weapon used				1.82 (0.36–9.31)
	Intoxicated				2.33 e-08 (0 -.)
	Number of offences				0.99 (0.67–1.47)

## Discussion

This study explored the association of circumstances during the index offence and victims’ characteristics with the frequency of engagement in self-harming behaviors among forensic patients with a violent index offence. The results showed that of the overall 24.06% explained by the models, the victim’s characteristics contribute approximately 5% points, and circumstances during the index offence contribute an additional 2% points in explaining self-harming behaviors among forensic psychiatric patients during the reporting year. The only factors associated with an increase in the risk of self-harming behaviors from the study hypotheses were if the victim was a healthcare/support staff or a co-patient/cohabitant.

Multiple self-harming behaviors (61.7%) in the present study were much higher than the 48.1% observed in a prospective study covering eleven years among forensic patients in the Netherlands [9]. Notably, this is the only study based on our literature review that presented results on multiple self-harming behaviors. The difference in the findings regarding our research and the study in the Netherlands may be attributed to the finding that most of the 113 self-harming incidents in the present study were primarily driven by one individual who had 44 incidences in one year. Additionally, another factor contributing to the disparity might be the study design; the Dutch study was prospective, which allowed for active management of individuals with a higher risk of re-engaging in self-harm as their behaviors were tracked throughout the study.

As evidenced in various studies, our results also showed that a history of self-harm is a major predictor of self-harm incidents [16, 39, 40]. Also, our findings showed that as forensic patients get older, there is an associated decrease in self-harming incidents. This reduction may be linked to the various physical health issues that often arise with age, which can reduce the likelihood of engaging in self-destructive behaviors. As patients age, they also gain life experience that helps them better understand the consequences of self-harm, encouraging the adoption of healthier coping mechanisms. Furthermore, as patients age within the forensic system, the care teams may develop more effective interventions for managing self-harm behaviors among these individuals.

When it comes to the study’s proposed hypothesis, it is essential to note that apart from clinical characteristics, victim characteristics emerged as the second most important factor in explaining the variation in the incidences of self-harming among forensic psychiatry patients. This emphasizes the significant contribution played by the victim characteristics (especially when the violence was perpetrated against healthcare staff, support worker, or co-patient) in understanding self-harming behaviors statistically. The nature of this association may reflect that the negative emotions, such as guilt and shame triggered in patients with violent offences, can vary depending on the victim’s characteristics.

Having a healthcare worker or support staff as a victim may be daunting to patients since they continue to see and work with healthcare workers throughout their period in the forensic system. This experience may make many feel guilty and ashamed of violently attacking a healthcare worker. This reality might be stressful for the affected patients, and some of them end up adopting maladaptive coping styles, which may involve self-harming behaviors. For such patients, compassion-focused models may be beneficial in their management to help them adjust to the reality of living with constant triggers or stressors related to the identity of the victim [41, 42]. Healthcare workers must be cognizant of the potential complications of reminding such patients about their index offence or triggering stressful memories and help them adjust to working with other health workers. To assist health workers in navigating these challenges of dealing with patients who violently attacked them or a colleague(s), we suggest creating training programs specifically designed for their needs. These programs should aim to equip healthcare professionals with essential skills for managing complex cases to prevent future similar incidences and increase confidence, grasp the psychological factors behind violent actions, and employ evidence-based interventions to manage the situation [43, 44]. Furthermore, for health workers affected by these distressing events, offering tailored therapy could prove immensely helpful in aiding their recovery [45] Also, suppose the risk of self-harming behaviors remains high for patients in the same facility where the index offense was perpetrated; such patients may be beneficially transferred to another facility away from the staff they violently offended [11]. Another aspect to note is that attacking a staff member is usually perceived as a grave offense, potentially leading to differential treatment by staff, which could lead, in turn, to feelings of exclusion, loneliness, depressive symptoms, and self-harm. In such circumstances, it may be worth noting that transferring a patient who attacked a staff member could benefit the patient and the healthcare worker(s) [44].

Similarly, it may be emotionally challenging to cope with the anger, guilt, shame, or frustration of engaging in a violent attack on a co-patient. Following these emotions, the affected patients might resort to using self-harm as a way of coping, expressing, or regulating these emotions, even though it is a maladaptive coping mechanism [21, 23]. The presence of active psychotic symptoms, such as paranoid delusions towards co-patients or individuals with mental illness, may worsen the feelings associated with assaulting a co-patient [46]. Considering the vulnerability of patients with mental illness, as the insight of the patients who perpetrated violent offences improves, they may start to feel ashamed and guilty for having attacked their victim, thus perpetuating self-harming behaviors in response [9]. In addition, the patients may develop paranoia about co-parents planning revenge, which could contribute to hypervigilance, emotional unrest, and subsequent self-harming behaviors as an attempt to cope with the emotions above.

Depending on the relationship between the patient and victim, varying emotional responses may be triggered in various individuals, leading to varying coping strategies. Unfortunately, some patients may consider coping with such negative emotions by using self-harming. A better understanding of the link between victim relationships and self-harming may require prospective studies with qualitative designs focusing on exploring the impacts of these relations. Interestingly, the current study provides a starting point in explaining and understanding this relationship.

On a broad note, the study’s findings raise important questions regarding the potential link between victims’ impact statements during the ORB hearings and self-harming behaviors, especially since the factors related to the severity of the physical injury were not associated with self-harming behaviors. Overall, this aspect requires further investigations to dive deep into the relationship between self-harming behaviors and factors that are related to how individuals who perpetrated violent offences handle being confronted with the consequences of their offences on the victims, which may include negative impacts on their emotional well-being.

Although the study hypothesis postulated a potential link between guilt, shame, and regrets about the circumstances surrounding the index offence and self-harm incidents, our results indicated these specific factors did not have a significant contribution to the individual’s risk of self-harming. Specifically, the results suggest that the use of a weapon, intoxication, or perpetration of numerous offences did not affect the risk of patients engaging in self-harming behaviors. This could be due to the study design focusing on statistical associations instead of patients’ perceptions. A qualitative study might provide a clearer understanding of this relationship. Additionally, the timing of the assessment in relation to the index offense may influence the results. Shortly after the index offense, patients might experience greater feelings of shame and guilt compared to years later. However, it is essential to note that despite controlling for time since the index offence in the present study, a similar study assessing the influence of these factors on self-harm among individuals who have been in the forensic system for less than a year may yield different findings. Emotional response directly following the offence may be more intense in many individuals compared to the reality in this study, which studied many participants who have spent substantial periods in the system.

### Limitations and recommendations

The study has several limitations, such as the retrospective design, the reliance on the recorded and available data only, and the missing information on some variables. Overall, the factors included in the model are not exhaustive. For example, relevant information on the methods of self-harming behaviors, the nature of weapon use, and total incidents of self-harming behaviors, among others, were unavailable. Some of the missing or unavailable data would provide a better perspective in understanding the relationship of self-harm with violent offences, circumstances during the index offence, and victims’ characteristics. We, therefore, recommend that future studies utilize a longitudinal study design and capture relevant information to address the highlighted limitations and allow an exploration of the causal mechanisms, including the trend or patterns of the relationship between weapon use and self-harm among violent offenders, taking into consideration the effect of time. We also used the listwise deletion method in our analysis, which reduced the statistical power due to the presence of missing data in our dataset and impacts the generalizability of the findings. Despite most potential explanations being related to negative emotions such as shame and guilt, the present study did not capture information about shame and guilt. We recommend future studies to explore the mediating role of these factors in self-harming behaviors and use methods such as structure equation modelling. Other potential aspects missed due to the nature of the data used were the lack of consideration of important historical characteristics that are key in management, such as the age of the first self-harming incident and previously used management methods. We recommend future studies to capture and analyze these important aspects. Also, despite double data entry and supervision of the process, we did not have any statistical analysis to determine the reliability of the data entry process. We recommend that future researchers calculate the interrater reliability using measures such as Kappa statistics. Lastly, given the relatively small sample sizes in some subgroups, it is important to acknowledge its potential impact on our findings’ statistical power. This limitation may affect the generalizability and robustness of the results, and caution should be exercised when interpreting the data. Future research should aim to include larger and more diverse multinational sample sizes to improve the statistical power and reliability of the findings.

### Implications

This study advances current knowledge on violence and self-harm among forensic psychiatric patients and has important implications for clinical practice and policymaking. The study reveals that self-harm is a common and multifaceted behavior in forensic populations with violent offences, and the risk of harm to self can be shaped by psychological responses that are related to various factors, including the patient’s background, mental health, offence, and victim’s related attributes. The study indirectly supports the guilt and shame hypothesis, which suggests that self-harm might be a maladaptive way of coping with negative emotions triggered by harming others [23, 25]. The study calls for comprehensive and individually tailored interventions, addressing vulnerabilities for shame, guilt, emotion dysregulation, mental health issues, and negative emotions related to the victim to prevent and mitigate self-harm among forensic patients. The study also adds to the existing literature underpinning the potential role of the shame and guilt hypothesis in explaining self-harm among the forensic population. Clinicians and other stakeholders in the forensic psychiatry system need to be cognizant of these findings in clinical practice and policymaking.

## Conclusion

This study reveals the multifaceted nature of self-harm among forensic patients who have committed violent offences. Contrary to our expectations, circumstances surrounding the index offence did not significantly explain the variance in the incidents of self-harm. Instead, we found that victim characteristics, such as their relationship with the offender, were associated with self-harm. It may be that these specific factors confer a greater sense of shame and guilt, which may then translate into greater engagement in self-harming behaviors. The results highlight the need for comprehensive and individually tailored interventions to prevent and reduce self-harm among this vulnerable population.

## Data Availability

Due to the sensitivity of the population being explored, the datasets will be made available to appropriate academic parties on request from the corresponding author after approval by GAC.
